# First case report of spontaneous biliary pleural fistula diagnosed using near infrared region I/II fluorescence of indocyanine green

**DOI:** 10.3389/fonc.2022.906812

**Published:** 2022-08-05

**Authors:** Yisheng Peng, Jun Fan, Gang Zhu, Cheng Fang, Fangyi Peng, Zeyu Zhang, Jie Tian, Song Su, Xiaoli Yang, Bo Li

**Affiliations:** ^1^ Department of General Surgery (Hepatobiliary Surgery), The Affiliated Hospital of Southwest Medical University, Luzhou, China; ^2^ Nuclear Medicine and Molecular Imaging Key Laboratory of Sichuan Province, The Affiliated Hospital of Southwest Medical University, Luzhou, China; ^3^ Academician (Expert) Workstation of Sichuan Province, The Affiliated Hospital of Southwest Medical University, Luzhou, China; ^4^ Key Laboratory of Molecular Imaging, Institute of Automation, Chinese Academy of Sciences, Beijing, China

**Keywords:** biliary pleural fistula, NIR fluorescence, ICG, diagnosis, case report

## Abstract

We report a rare case of spontaneous biliary pleural fistula in a patient whose diagnosis was aided by the use of near-infrared I/II fluorescence imaging. When both 99mTc-mebrofenin hepatobiliary scintigraphy and CT examination were diagnostically difficult, we found strong fluorescent signals in the patient’s pleural drainage fluid and sputum using NIR I/II fluorescence imaging, and therefore diagnosed the patient with a biliary pleural fistula. This provides a safe and effective test for diagnosing biliary pleural fistulas.

## Introduction

Biliary pleural fistula is a pathological traffic between the biliary system and the pleura, through which bile enters the pleural cavity and travels as a biliary pleural effusion, and is one of the serious complications of biliary disease (1). Because of its insidious symptoms and clinical rarity, it is highly susceptible to misdiagnosis and underdiagnosis, and its mortality rate may exceed 50% (2). The main causes of biliary pleural fistulae are amoebic liver abscess, hepatic peritonitis, post-radiofrequency ablation, trauma, post-pneumonectomy, and complications after hepatectomy (3, 4). The common tests currently used for biliary pleural fistula include 99mTc-mebrofenin hepatobiliary scintigraphy, cholangiography, CT of the chest and abdomen, and MRCP (5–7). However, cholangiography may trigger risks such as cholangitis and radiation in patients. CT and MRCP have low diagnostic efficacy for microfistulae and are highly likely to be missed (8).

Near-infrared window I (NIR-I, 700-900 nm) fluorescence imaging has good spatial and temporal resolution and has been widely used in clinical applications such as: real-time intraoperative fluorescence navigation and diagnostic imaging (9). Near Infrared Window II (NIR-II, 1000-1700 nm) fluorescence imaging offers better imaging quality and its sensitivity is higher (10). Indocyanine green is widely used in the preoperative assessment of liver function. It is first bound to plasma proteins in the blood, then taken up by the parenchymal cells of the liver and finally excreted from the biliary system *via* the bile (10, 11).

However, so far, there is no research on the near-infrared window region I/II fluorescence technique for diagnosing biliary pleural fistula. Therefore, this study aimed to evaluate the feasibility and effectiveness of the near-infrared window region I/II fluorescence technique in the diagnosis of biliary pleural fistula.

## Case report

**Figure 1:**
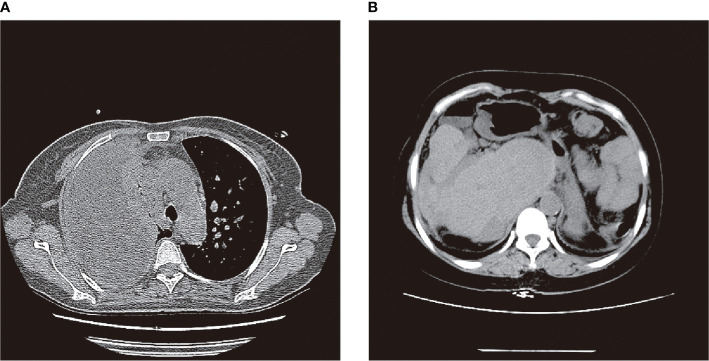
CT examination: **(A)** patient with massive right pleural effusion and inflammatory lesions; **(B)** post-hepatectomy, post-splenectomy, severe liver deformation, intrahepatic bile duct stones and dilated intrahepatic bile ducts.

**Figure 2:**
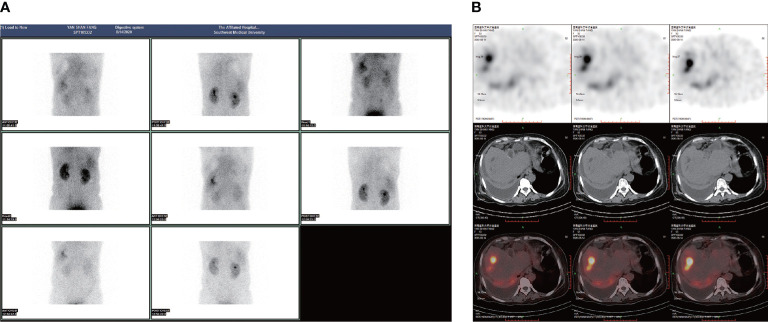
99mTc-mebrofenin hepatobiliary scintigraphy: **(A, B)** Impaired liver function and large right pleural effusion with no obvious developer distribution within it.

**Figure 3:**
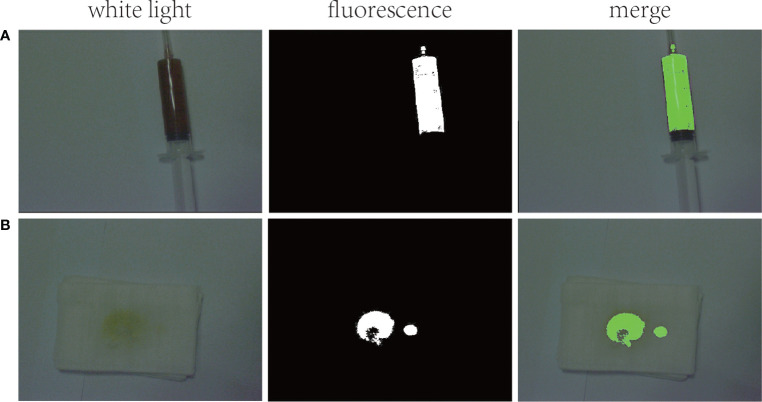
Fluorescence imaging in the NIR I region: **(A)** strong fluorescent signal seen in pleural effusion; **(B)** strong fluorescent signal seen in sputum.

**Figure 4:**
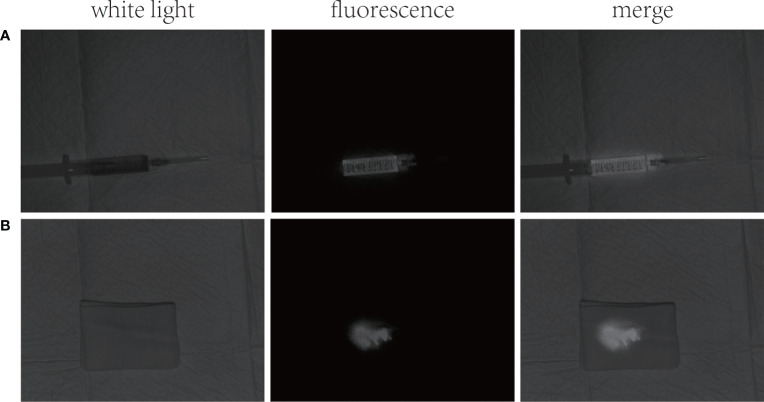
NIR region II fluorescence images: **(A)** strong fluorescent signal in pleural effusion; **(B)** strong fluorescent signal in sputum.

A 53-year-old female was admitted to the hospital on 18/08/2021 with “yellowish staining of the skin and sclera with dyspnea after activity for 20+ days”. Past history: 9+ years ago, he underwent the combined splenectomy with left lobe resection of the liver for calculus of bile duct and 1+ years ago, he underwent endo scopic haemostasis for cirrhosis with ruptured oesophagogastric fundic varices. The patient presented two days after admission with worsening dyspnea and coughing up of yellowish-brown sputum. The patient’s serum bilirubin was 316 umol/L, and the thoracentesis showed yellow-green pleural effusion, and the pleural effusion examination showed a mildly elevated bilirubin level of 76 umol/L. The patient was then suspected of having a possible biliary pleural fistula. Therefore, CT examination (**Figure 1**) and 99mTc-mebrofenin hepatobiliary scintigraphy (**Figure 2**) were performed and no significant fistulae were found. Indocyanine green (0.5mg/kg) was then injected intravenously and the patient waited for 24 hours. Pleural effusion and sputum were collected again and the presence of fluorescent signals in them was detected using a near-infrared I-region fluorescence imaging system provided by the Beijing Key Laboratory of Molecular Imaging (Digital Precision Medicine). Interestingly, we can clearly observe a strong fluorescent signal in both pleural effusion and sputum (**Figure 3**). Also, we found strong fluorescent signals in pleural effusions and sputum using near-infrared region II fluorescence imaging (**Figure 4**). Therefore, the patient was considered to be diagnosed with a biliary pleural fistula. We recommend that the patient is dissected for a definitive diagnosis of a biliopleural fistula and that a repair of the fistula hole is performed if available. Unfortunately, after assessment of the patient’s general condition and multidisciplinary consultation and discussion, it was concluded that the patient’s current poor general underlying condition, with liver failure, severe lung infection and severe morphological deformation of the liver, made surgical treatment an extremely high risk and endoscopic nasobiliary drainage (ENBD) treatment could be considered. After communication with the patient and his family, they refused to undergo further investigations and treatment due to the risks of surgery and financial reasons. After 20 days in hospital, the patient did not improve significantly and the sufferer chose to abandon treatment. The patient unfortunately passed away after 1 month of follow-up.

## Discussion

Fluorescence imaging following intravenous injection of ICG has already been used widely for cholangiography (12). Compared with the first near-infrared (NIR-I) optical window (650-950 nm), the fluorescence imaging of the second near-infrared optical window (NIR-II, 950-1700 nm) has better tissue penetration, with facilitates *in vivo* deep tissue imaging and real-time localization of tumors (13). NIR II imaging is widely used in surgery, such as real-time guided liver cancer resection, lung tumor resection, lymph node dissection, photothermal therapy, etc (10). It has good application prospects.

In this case, we consider a spontaneous biliary pleural fistula caused by a long-term diffuse intra- and extra-hepatic bile duct stone causing biliary obstruction, which raised the biliary pressure and then formed a leak of hepatic peritoneum into the thoracic cavity. The biliary pleural fistula in this patient may be very small, so neither the 99mTc-mebrofenin hepatobiliary scintigraphy nor CT examination found the existence of the patient’s fistula. At the same time, the bilirubin level in the patient’s pleural effusion was only slightly elevated and did not reach the level (triple) for diagnosing bile leakage (14). Therefore, it is difficult to judge whether it is a biliary pleural fistula from the color and nature of the pleural effusion. We considered arranging a cholangiogram to definitively diagnose a biliopleural fistula. However, the patient declined to undergo cholangiography due to his poor general underlying condition. It is currently difficult to diagnose this patient with a biliary pleural fistula. At this time, there is a great need to find a new and safe method to aid in the diagnosis of biliary pleural fistula. One study reported the successful application of ICG fluorescence technique for the diagnosis of biliary leakage during hepatectomy (15). Therefore, for the first time, we successfully assisted in the diagnosis of the presence of bile in both the pleural fluid and sputum of this patient using near-infrared region I/II fluorescence imaging based on the metabolic characteristics of ICG. In the patient’s pleural fluid and sputum, we can clearly observe the presence of strong fluorescent signals. It shows that NIR region I/II fluorescence imaging is highly sensitive and that it is very easy and safe to use. Furthermore, NIR Region I/II fluorescence imaging avoids the risk of exposing the patient to radiation and allows for diagnosis through a non-invasive approach. This provides another new way to diagnose biliary pleural fistulas.

However, the location of the fistula in the abdominal cavity is difficult to detect due to the weak penetration of NIR region I/II fluorescence imaging. In the future, if ICG fluorescence and 99mTc-mebrofenin hepatobiliary scintigraphy are combined, this will further improve the diagnosis of biliopleural fistulas.

Near-infrared region I/II fluorescence imaging is a new method for the diagnosis of biliary pleural fistulas, which has the advantage of being safe, convenient and sensitive. It has very promising applications in the future to assist in the diagnosis of biliary pleural fistulae.

## Data availability statement

The original contributions presented in the study are included in the article/supplementary material. Further inquiries can be directed to the corresponding authors.

## Ethics statement

The studies involving human participants were reviewed and approved by Ethics Committee of Affiliated Hospital of Southwest Medical University. The patients/participants provided their written informed consent to participate in this study. Written informed consent was obtained from the individual(s) for the publication of any potentially identifiable images or data included in this article.

## Author contributions

BL and XY conceived the idea of the project. YP, JF and GZ wrote the manuscript in addition to designing, performing all experiments. CF and FP performed the experiments. ZZ and JT collected the information on patients with biliary pleural fistulae. SS assisted with experimental design, manuscript preparation and image analysis. BL and XY designed, supervised and analyzed all experiments, in addition to assisting with manuscript preparation. All authors contributed to the article and approved the submitted version.

## Funding

This work was supported by Key Research and Development Project of the Science & Technology Department of Sichuan Province (Nos.2021YFS0231, 22ZDYF1898), China Postdoctoral Science Foundation Funded Project (Grant Nos. 2020M673096).

## Conflict of interest

The authors declare that the research was conducted in the absence of any commercial or financial relationships that could be construed as a potential conflict of interest.

## Publisher’s note

All claims expressed in this article are solely those of the authors and do not necessarily represent those of their affiliated organizations, or those of the publisher, the editors and the reviewers. Any product that may be evaluated in this article, or claim that may be made by its manufacturer, is not guaranteed or endorsed by the publisher.
